# Exploring the Impact of 3D Fast Spin Echo and Inversion Recovery Gradient Echo Sequences Magnetic Resonance Imaging Acquisition on Automated Brain Tumor Segmentation

**DOI:** 10.1016/j.mcpdig.2024.03.006

**Published:** 2024-04-16

**Authors:** Mana Moassefi, Shahriar Faghani, Sara Khanipour Roshan, Gian Marco Conte, Seyed Moein Rassoulinejad Mousavi, Timothy J. Kaufmann, Bradley J. Erickson

**Affiliations:** a Mayo Clinic Artificial Intelligence Laboratory, Department of Radiology, Mayo Clinic, Rochester, MN; b Department of Radiology, Mayo Clinic, Rochester, MN

## Abstract

**Objective:**

To conduct a study comparing the performance of automated segmentation techniques using 2 different contrast-enhanced T1-weighted (CET1) magnetic resonance imaging (MRI) acquisition protocol.

**Patients and Methods:**

We collected 100 preoperative glioblastoma (GBM) MRIs consisting of 50 IR-GRE and 50 3-dimensional fast spin echo (3D-FSE) image sets. Their gold-standard tumor segmentation mask was created based on the expert opinion of a neuroradiologist. Cases were randomly divided into training and test sets. We used the no new UNet (nnUNet) architecture pretrained on the 501-image public data set containing IR-GRE sequence image sets, followed by 2 training rounds with the IR-GRE and 3D-FSE images, respectively. For each patient, in the IR-GRE and 3D-FSE test sets, we had 2 prediction masks, one from the model fine-tuned with the IR-GRE training set and one with 3D-FSE. The dice similarity coefficients (DSCs) of the 2 sets of results for each case in the test sets were compared using the Wilcoxon tests.

**Results:**

Models trained on 3D-FSE images outperformed IR-GRE models in lesion segmentation, with mean DSC differences of 0.057 and 0.022 in the respective test sets. For the 3D-FSE and IR-GRE test sets, the calculated *P* values comparing DSCs from 2 models were .02 and .61, respectively.

**Conclusion:**

Including 3D-FSE MRI in the training data set improves segmentation performance when segmenting 3D-FSE images.

Glioblastoma multiforme (GBM), the most common primary malignant brain tumor in adults, accounts for 77%-81% of all primary malignant tumors of the central nervous system.^1^ More than 10,000 Americans could have been diagnosed with GBM in 2023 based on a 3.19-per-100,000 incidence rate.^2^ Magnetic resonance imaging (MRI) provides detailed brain structure for diagnosing GBMs. On MRI, GBM tumors are located and semantically segmented, and the tumor size is measured. The findings derived from this process enable radiologists and clinicians to determine the tumor components, size, and aggressiveness and plan further treatment, such as surgical and radiation therapy.^3^

In contrast to some tumors with more homogeneous structures, like meningiomas, GBMs are diffusely infiltrative, heterogeneously enhancing, and frequently centrally necrotic and have asymmetrical shapes and extended tentacle-like structures, making them more complex and controversial to delineate.^4^ Moreover, unlike computed tomography scans, MRI does not possess a standardized or absolute voxel value scale to allow thresholding. Variations in scanners (field strength of 1.5, 3, or 7 T) and image acquisition protocols (eg, field of view, voxel resolution, gradient strength, and several other parameters such as repetition and echo times [TE]) can result in substantial differences in grayscale values of the same tumor across different imaging centers. These factors complicate automatic tumor segmentation. Therefore, expertise and considerable time and cost are necessary for manual GBM segmentation on MRI.

Automated GBM segmentation using deep learning models offers a cost-efficient solution that significantly reduces the time required for this time-consuming task.^5^ This approach can be implemented with or without the further supervision of radiologists, providing a consistent and efficient workflow.^6^ By using the same algorithm for all cases, the segmentation process becomes more consistent compared with the variability introduced by multiple radiologists’ opinions. Accurate segmentation plays a crucial role in radiation planning, response assessment across multiple examinations, and the evaluation of specific tumor regions using advanced imaging techniques such as perfusion and diffusion parameters within contrast-enhancing portions of the tumor.^7^ Performing GBM tumor segmentation using automated deep learning–based tools has shown great promise in recent years.^8^, ^9^, ^10^, ^11^, ^12^ In 2021, van Kempen et al^11^ found 42 studies reporting deep learning–based glioma segmentation models. Currently, only 1 tool has received the US Food and Drug Administration (FDA) approval for this task.^13^

In the context of lesion detection and characterization using MRI, diverse techniques and pulse sequences are available, each possessing distinct inherent attributes that allow the highlighting of particular aspects of different tumors’ pathology. The radiologists’ expertise relies on using different magnetic resonance sequences to best depict these various aspects of tumors and other diseases. Patients with GBM routinely undergo a standard brain tumor MRI protocol, including fluid-attenuated inversion recovery (FLAIR), T1 weighted image (WI), T2WI, and contrast-enhanced T1-weighted (CET1) MRI.^14^ CET1 is a crucial sequence that provides essential insights into tumor location, degree of enhancement and aggressiveness, edema, and necrotic core. The conspicuity of contrast-enhancing lesions (CELs) in relation to the background parenchyma is affected by several factors, including the ultrastructural features of the blood-brain barrier, magnetic field strength, concentration of gadolinium-based contrast agents, relaxivity properties, time elapsed since injection, and MRI acquisition technique.^15^, ^16^, ^17^, ^18^

Over the past decade, inversion recovery gradient echo (IR-GRE) techniques, such as the 3-dimensional (3D) T1 Magnetization Prepared - RApid Gradient Echo, have emerged as popular pulse sequences for CET1 MRI. These 3D, thin-section techniques can be reformatted into various planes and leverage the inversion recovery preparation pulse to emphasize the contrast between gray and white matter in the brain.^19^^,^^20^ IR-GRE techniques such as Magnetization Prepared - RApid Gradient Echo have been recommended for use in brain tumor clinical trials by the Consensus Recommendations for a Standardized Brain Tumor Imaging Protocol in Clinical Trials and the modified Response Assessment in Neuro-Oncology criteria,^14^^,^^21^ depicting anatomy very well with excellent signal-to-noise ratio and spatial resolution. However, compared with T1-weighted 3D fast spin echo (FSE) techniques such as CUBE, Sampling Perfection with Application-optimized Contrast using different flip-angle Evolutions (SPACE), and volume isotropic turbo spin echo acquisition (VISTA), IR-GRE images generally yield poorer conspicuity of postgadolinium CELs, particularly if those lesions are within the relatively bright white matter of IR-GRE (which contributes to diminished contrast-to-noise ratio [CNR] of CELs) or if near the enhancing arterial and venous vessels of IR-GRE.^22^, ^23^, ^24^, ^25^ This limitation can hinder the accurate detection of lesions, particularly those with subtle contrast enhancement.

Mugler et al^26^ developed the SPACE sequence as part of their efforts to improve the quality of MRI imaging. SPACE, a 3D-FSE sequence, features variable flip-angle refocusing pulses and can be performed at 3 or 1.5 T. Compared with other techniques, the 3D-FSE method used by SPACE offers greater sensitivity to gadolinium enhancement, particularly in white matter where the CNR of CELs is increased.^24^^,^^25^ It can better withstand the negative impact of tumoral hemosiderin and calcium deposits, which can hinder the visualization of enhancement^27^ (Figure 1). As a “black blood” technique that largely suppresses signals related to gadolinium within blood vessels, 3D-FSE improves the conspicuity of small enhancing lesions in the brain periphery near subarachnoid vessels.^25^^,^^28^, ^29^, ^30^

**Figure 1:**
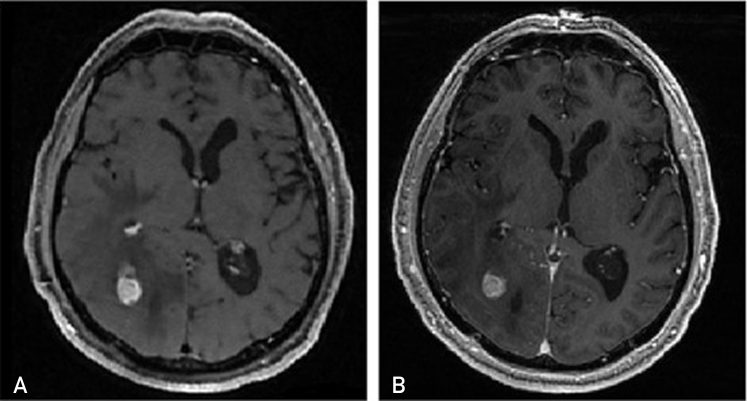
(A) A tumor with contrast-enhancing lesions using a 3-dimensional fast spin echo magnetic resonance imaging (MRI). (B) The same tumor and time point using an inversion recovery gradient echo MRI.

The successful segmentation of components on MRI is contingent on accurately differentiating their associated signals. To achieve this, it is common practice to use multiple MRI sequences (FLAIR, T1WI, T2WI, and CET1), which provide varying tissue contrasts, for GBM segmentation. Although studies have demonstrated that manual segmentation using 3D-FSE and IR-GRE can result in discrepancy among enhancing lesion components, to our knowledge, no research has assessed how automated segmentation models differentiate between these areas when using IR-GRE or 3D-FSE.^17^ Given the escalating utilization and advantages of 3D-FSE, we conducted this study to address this knowledge gap.

## Patients and Methods

This retrospective study was reviewed and exempted from the requirement for institutional review board approval. We conducted a series of experiments to assess the impact of training and testing segmentation models on CET1 images acquired with IR-GRE or 3D-FSE pulse sequence protocols.

### Data Set

We used 3 distinct data sets: the publicly available University of California San Francisco Preoperative Diffuse Glioma MRI (UCSF-PDGM) data set containing multiple pulse sequences and associated segmentation masks for patients with GBM.^31^ This data set comprises 501 cases with T1WI, T2WI, CET1, and FLAIR pulse sequences. The CET1 image sets in this data set are acquired using the IR-GRE protocol.^31^

The other 2 private data sets were collected from the Mayo Clinic institutional imaging database. We selected 100 patients with GBM diagnosed with isocitrate dehydrogenase wild-type tumors after January 1, 2018. We collected each patient’s preoperative FLAIR, T1WI, T2WI, and CET1 MRIs. Both data sets contained T1WI, T2WI, and FLAIR images with similar protocols. However, 50 patients had CET1 acquired using the IR-GRE acquisition protocol, and the other 50 patients had CET1 acquired using the 3D-FSE acquisition protocol.

In the IR-GRE acquisition protocol, the TE was observed to range from 1.86 to 23 milliseconds, having a median value of 3.05 milliseconds. The repetition time extended from 6.13 to 2300 milliseconds, with the median being 400 milliseconds. The inversion time ranged from 450 to 100 milliseconds, with a median of 900 milliseconds. The 3D-FSE acquisition protocol was used, where the TE varied between 8.15 and 44 milliseconds, with a median value of 12.65 milliseconds. The repetition time spanned from 400 to 1959.76 milliseconds, with a median of 600 milliseconds. For both protocols, MRI scanners with magnetic field strengths of both 3 and 1.5 T were used. Both the IR-GRE and J3D-FSE image sets contained scans with slice thicknesses ranging from 3 to 5 mm. We selected image sets with dimensions of 512 × 512 for the x-axis and y-axis and with slice counts ranging from 24 to 60.

For making the gold-standard segmentation mask for 100 Mayo Clinic image sets, a primary set of masks were computed using a publicly available glioma segmentation model (HD-GLIO; https://github.com/NeuroAI-HD/HD-GLIO-AUTO) and then manually reviewed by a neuroradiologist (B.J.E.) with 29 years of experience and a neuroradiology fellow in training from our institution (S.K.R.).^32^^,^^33^ The data sets consisted of 3 distinct mask classes, wherein the class denoted “0” referred to the background, “1” denoted edema, and “2” denoted CEL. Because the primary distinction between 3D-FSE and IR-GRE lies in their contrast enhancement, we kept the focus of our segmentation and performance comparison analysis on the CEL class.

We divided data sets into training and test sets. Because the USCF data set was used for pretraining, it was subsequently split into training and validation sets, with 450 samples allocated to the training set and 51 to the validation set. The 2 private data sets (1 with 50 patients having IR-GRE and 1 with 50 patients having 3D-FSE) were divided into 35-patient training sets and 15-patient test sets. The FLAIR and CET1 pulse sequences were selected for model training and testing from a pool of collected sequences. Using T2 and T1 sequences in the model did not yield improvement in results and impeded the training process.^34^^,^^35^ The HD-GLIO-AUTO was used to coregister each patient’s FLAIR and CET1 sequences in the Mayo data set, whereas the UCSF data set had already undergone coregistration and preprocessing.

### Model

We used no new UNet (nnUNet) to train our models.^36^ The nnUNet is a deep learning–based segmentation model that has the capability to customize itself for various tasks in the biomedical field automatically. This includes configuring the preprocessing, network architecture, training, and postprocessing stages. The process relies on a combination of fixed parameters, interdependent rules, and empirical decisions to make key design choices. With nnUNet, there is no need for manual hyperparameter tuning. Presently, nnUNet is the leading-edge tool for segmentation tasks.^33^^,^^37^ Fundamentally, a 3D UNet lies at the heart of the nnUNet model, which processes patches that are 128 × 128 × 128 in size. The network comprises an encoder-decoder architecture that connects the 2 pathways through skip connections. The encoder includes 5 levels of convolutional layers with the same resolution and strided convolution downsampling. The decoder follows the same design, with transpose convolution upsampling and convolution applied to concatenated skip features from the encoder branch at the same level. After each convolution operation, a leaky rectified linear unit with a slope of 0.01 and batch normalization was used. The FLAIR and CET1 volumes were concatenated and used as 2-channel input.

### Training

We applied nnUNet version 1 for 1000 epochs to each training set. We used the stochastic gradient descent method with an initial learning rate of 0.01 and with a batch size of 2. To enhance generalization, we applied data augmentation during training. This was done on the fly and included techniques such as random rotation and scaling, elastic deformation, gamma scaling, mirroring, and additive brightness augmentation.

The first model was trained and validated on the UCSF-PDGM training set. In the next step, we used the weights of the previous models and fine-tuned the model with Mayo Clinic private data sets. We conducted the fine-tuning process twice: once using the data set containing 35 IR-GRE image sets and once using the data set containing 35 3D-FSE image sets. We used 5-fold crossvalidation for the models, and the best model for each fold was saved. We conducted the experiments with Pytorch 1.13. We used 4 NVIDIA A100 GPUs for our computational tasks. The training took an average of 90 seconds for each epoch.

### Performance Comparison

We used the mean dice similarity coefficients (DSCs) as the performance metric for the comparison analysis. DSC is a commonly used metric for evaluating the performance of a segmentation model in medical image analysis, computer vision, and other fields where segmentation is used.^38^ It measures the similarity between the predicted segmentation mask and the ground truth segmentation mask. The formula for the DSC is as follows:

2×(intersection)/(union+intersection)

where intersection refers to the number of pixels classified as positive by the model and ground truth; union refers to the number of pixels classified as positive by the model, ground truth, or both. The dice score ranges from 0 to 1, with 1 indicating perfect agreement between the predicted and ground truth masks.

We also computed the 95th percentile Hausdorff distance (HD95), which is a variant of the traditional Hausdorff distance. While the Hausdorff distance measures the maximum distance between the boundaries of 2 sets (such as ground truth and predicted masks), HD95 considers only the largest 95% of these distances. This adjustment makes HD95 less sensitive to outliers than the standard Hausdorff distance. Specifically, it calculates the distance within which 95% of all points from one boundary lie from the nearest point on the other boundary. This metric provides a more robust measure of spatial accuracy in segmentation by focusing on most boundary points and offering a realistic view of the precision of object contours. HD95 is particularly useful for assessing segmentation accuracy because it complements metrics like the DSC.

### Statistical Analyses

**Figure 2:**
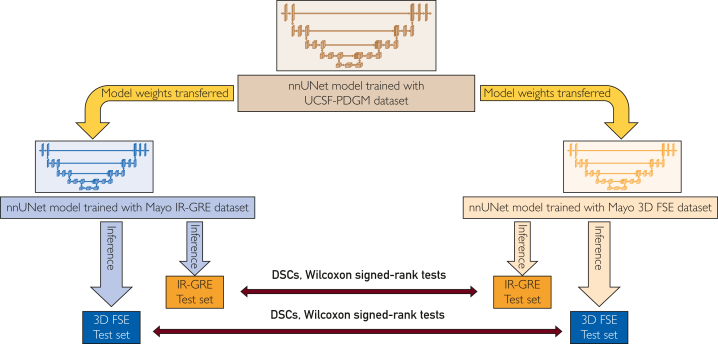
The pipeline of the study. IR-GRE, inversion recovery gradient echo; nnUNet, no new UNet; 3D-FSE, 3-dimensional fast spin echo; DSC, dice similarity coefficient; UCSF-PDGM, University of California San Francisco Preoperative Diffuse Glioma MRI.

The statistical analysis was conducted using the SPSS software package, version 27.0. (IBM). Normal distribution assumptions were assessed using the Kolmogorov-Smirnov and Shapiro–Wilk tests. We calculated 2 different DSCs for each case in the 3D-FSE test set. One DSC was calculated using the gold standard and predicted the mask obtained from fine-tuning the model with the 3D-FSE training set, and the other DSC was calculated using the gold standard and predicted the mask obtained from fine-tuning the model with the IR-GRE training set. Similarly, for each case in the IR-GRE test set, we performed the same calculation and obtained 2 DSC values. Subsequently, we conducted the Wilcoxon test to compare the 2 DSCs for each case in the set. Owing to the nonnormal distribution of our data, we conducted a 2-tailed, nonparametric paired Wilcoxon signed-rank test for paired measurements. Figure 2 shows the study pipeline.

## Results

Table summarizes the DSCs for the CEL segmentation of the 2 models for each patient in the test set. To clarify, each of the 15 cases in every test set belonged to entirely distinct patients. For instance, IR-GRE-01 represents an entirely separate patient and exhibits a completely different lesion compared with 3D-FSE-01.

**Table:** Dice Scores Obtained From the Inference Process on 2 Distinct Test Sets Using 2 Trained Models

3D-FSE test set					IR-GRE test set				
Case No.	Dice similarity coefficient from the model trained with 3D-FSE images	95th percentile Hausdorff distance	Dice similarity coefficient from the model trained with IR-GRE images	95th percentile Hausdorff distance	Case No.	Dice similarity coefficient from the model trained with 3D-FSE images	95th percentile Hausdorff distance	Dice similarity coefficient from the model trained with IR-GRE images	95th percentile Hausdorff distance
3D-FSE-01	0.98657394	2.00	0.92796691	1.00	IR-GRE-01	0.919188422	1.00	0.26451549	37.00
3D-FSE-02	0.92114801	1.00	0.866511041	17.49	IR-GRE-02	0.923418145	1.00	0.915960046	1.00
3D-FSE-03	0.593035821	18.27	0.116941342	21.12	IR-GRE-03	0.940072501	1.00	0.964708401	1.00
3D-FSE-04	0.948219096	1.00	0.920096194	1.00	IR-GRE-04	0.920271072	1.73	0.926250265	1.00
3D-FSE-05	0.954590467	1.00	0.918892838	1.00	IR-GRE-05	0.943799187	1.00	0.934584069	1.00
3D-FSE-06	0.953783423	1.00	0.952771946	1.00	IR-GRE-06	0.840494408	38.53	0.92400654	1.00
3D-FSE-07	0.969573031	1.00	0.927997562	1.00	IR-GRE-07	0.937335806	1.00	0.961940193	1.00
3D-FSE-08	0.906564037	1.00	0.936158135	1.00	IR-GRE-08	0.93983324	1.00	0.954972532	1.00
3D-FSE-09	0.959106679	1.41	0.914656837	1.00	IR-GRE-09	0.864130435	1.73	0.884445599	2.00
3D-FSE-10	0.851646208	9.90	0.704100187	19.74	IR-GRE-10	0.905986022	1.00	0.934689899	1.00
3D-FSE-11	0.98318633	1.00	0.96032081	1.00	IR-GRE-11	0.935465106	1.00	0.927665312	1.00
3D-FSE-12	0.906616185	7.07	0.971174116	2.00	IR-GRE-12	0.953264108	1.00	0.915632347	1.00
3D-FSE-13	NAN^a^	NAN^a^	NAN^a^	NAN^a^	IR-GRE-13	0.836125874	6.78	0.711017647	21.00
3D-FSE-14	0.965717792	1.00	0.907016845	1.00	IR-GRE-14	0.941141344	1.00	0.949803021	1.00
3D-FSE-15	0.952668198	1.00	0.915657094	1.00	IR-GRE-15	0.942733403	1.00	0.965552733	1.00
Average of dice scores	0.925991043		0.869137986		Average of dice scores	0.918736555		0.897179597	
SD	0.107190941		0.276314885		SD	0.041902273		0.197989519	
Median of dice scores	0.953783423		0.918892838		Median of dice scores	0.937335806		0.926250265	

IR-GRE, inversion recovery gradient echo; NAN, not a number; 3D-FSE, 3-dimensional fast spin echo.

a The NAN case lacked any contrast-enhancing component in their tumor region that could be segmented.

**Figure 3:**
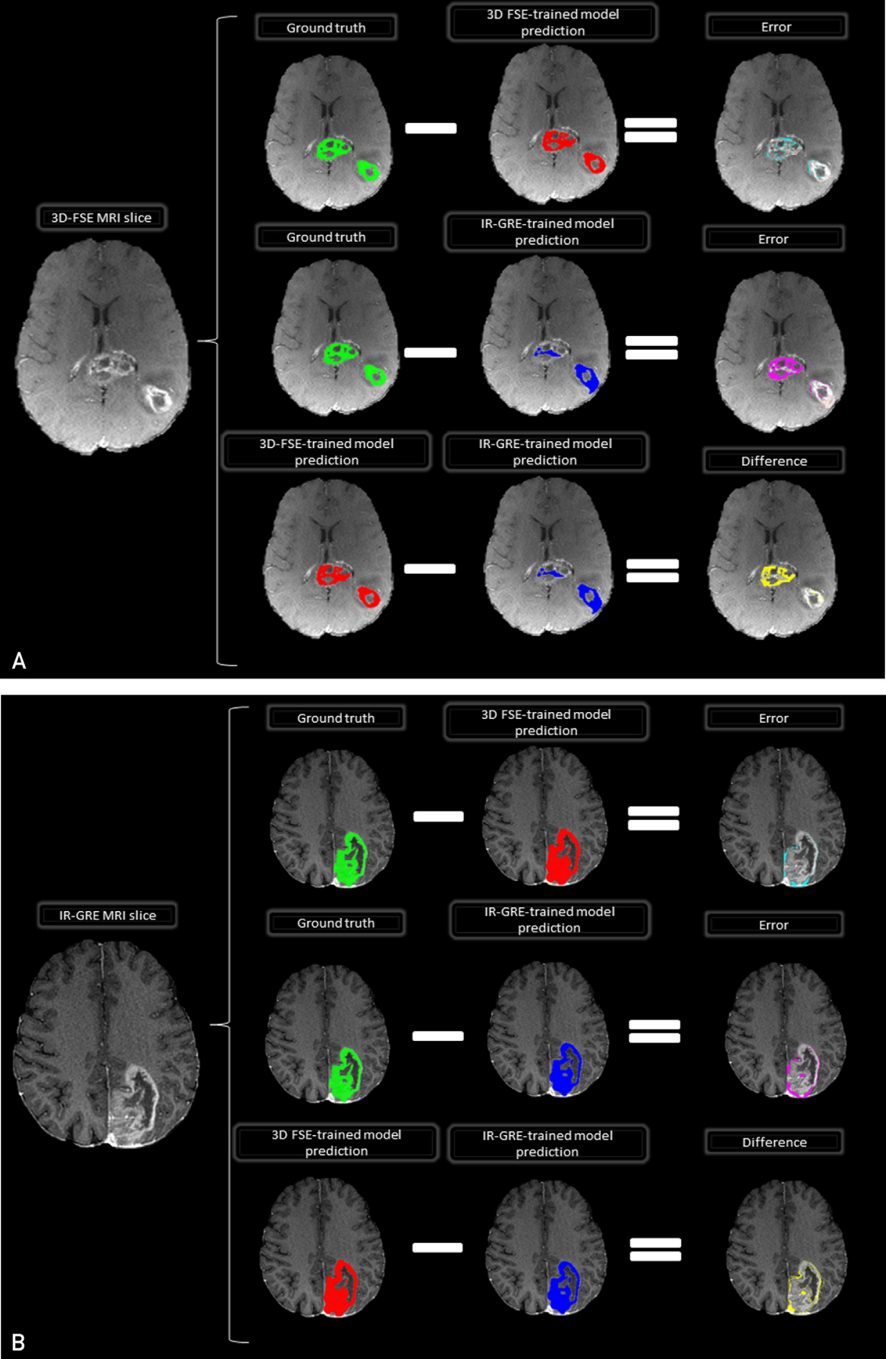
Two MRI slices showcasing a glioblastoma tumor lesion. (A) A 3D-FSE MRI slice is displayed, with colors representing the ground truth, predicted, error, and difference masks overlaid on the image. (B) An example of an IR-GRE MRI slice, depicting the ground truth, predicted, error, and difference masks using different colors. IR-GRE, inversion recovery gradient echo; 3D-FSE, 3-dimensional fast spin echo.

The calculated *P* values comparing 2 DSC sets using for cases in the 3D-FSE and IR-GRE test sets was .02 and .61, respectively. Figure 3 presents 2 MRI slices, highlighting a GBM tumor lesion on 3D-FSE and IR-GRE sequences.

## Discussion

In this study, we developed 2 distinct segmentation models, each specializing in the delineation of GBM lesions within either IR-GRE or 3D-FSE MRI protocol. Subsequently, we compared these models, evaluating their performance on separate test sets comprising cases obtained from the respective imaging protocols. Our findings reveal that the model specifically trained on the 3D-FSE protocol outperformed its counterpart in segmenting GBM lesions (even in the case of IR-GRE image sets). This superior performance was attributed to the heightened contrast visualization afforded by the 3D-FSE MRI protocol, enhancing the efficacy of lesion segmentation.

We followed the conventional practice of using a 70% to 30% split to partition our image data sets into training and test sets, respectively.^39^ Given our initial pretraining phase involving 450 cases, it was our supposition that including an additional 35 cases featuring different imaging protocols would sufficiently help the model learn the distinct features of each data set. Moreover, the incorporation of 15 cases for the precise computation of lesion-specific visual attributes diminished the likelihood of encountering stochastic or random outcomes in our study.

Regarding the results in Table, it is important to mention that, in a single instance (3D-FSE-13), the MRI scan did not exhibit any CEL components. Our data selection process deliberately omitted manual inspection to prevent any bias toward cases with more conspicuous lesions. Consequently, such occurrences were accommodated within the data set without bias, and we kept this case. Furthermore, our segmentation models encountered challenges in certain cases, such as 3D-FSE-03, where the lesions proved to be particularly intricate, posing difficulties for both models during the segmentation process.

Several research articles have described the superiority of 3D-FSE image acquisition in identifying small tumors and detecting subtle changes associated with CELs.^25^^,^^28^^,^^29^ First, Incorporating 3D-FSE sequences into the clinical imaging protocol for patients has a practical implication of improving the detection of regions with a more aggressive grade of malignancy within GBMs. This is crucial for accurately selecting biopsy samples, ensuring correct histologic grading, and making informed treatment decisions. Moreover, maximizing tumor resection has been linked to better patient outcomes, and image-guided approaches have the potential to increase the extent of resection.^40^ Therefore, optimizing the accuracy of target delineation enables precise surgical procedures tailored to individual patients. In the context of planning radiotherapy, the use of low-conspicuity images may hinder the delineation of target volume margins, resulting in suboptimal dose delivery to all regions of active tumor growth and/or unnecessary dose delivery to surrounding critical brain structures.^41^^,^^42^ As the utilization of automated segmentation continues to increase in GBM delineation clinical practice and 3D-FSE is becoming a popular sequence, it is crucial to determine whether training with this pulse sequence will impact tumor segmentation model performance or not.^17^

To our knowledge, this is the first study that conducted a comparative analysis of the impact of 3D-FSE in automated segmentation models in a general context. According to the findings of our study, models trained on 3D-FSE images demonstrate superior performance in segmenting CEL on both 3D-FSE and IR-GRE image sets. Specifically, we observed an increase of 0.057 in the average of DSCs for the test set cases with 3D-FSE image sets and 0.022 for the test set cases with IR-GRE image sets. We hypothesized that this is due to the superior CNR of the 3D-FSE images, which assists the model in learning the edges and detecting the lesions more effectively. By incorporating 3D-FSE image sets or performing a single step of fine-tuning, GBM segmentation models can be optimized and further refined, thereby improving their performance. Our findings have implications for medical centers with access to 3D-FSE capabilities and for the broader research community using publicly available GBM segmentation models, facilitating improved accuracy and clinical decision-making in the context of GBM segmentation and volumetric analysis.

In this study, we note that our model was pretrained on a data set consisting of 501 IR-GRE cases, which was necessary to achieve an acceptable level of performance for comparative analysis. The models trained solely on 50 cases, without the benefit of pretraining, yielded average DSCs of 0.68, which fell short of the desired threshold for establishing fair comparisons. Ideally, pretraining with the same number of 3D FSE cases would have been preferred for conducting a comprehensive comparative analysis. However, the unavailability of such a data set, coupled with the high costs associated with annotating 501 cases, made this impractical. Nonetheless, although not ideal, our approach of pretraining on 501 IR-GRE cases and subsequently fine-tuning on 50 3D FSE or IR-GRE cases demonstrated the potential benefit of incorporating a fine-tuning step with 3D FSE data.

A limitation of our study arose from our use of segments from 100 patients with GBM, primarily because of the significant time and cost associated with data annotation. Nonetheless, to address the challenge posed by a limited patient count, we initially conducted a pretraining step using a publicly available data set. In our future research efforts, we plan to expand our data set with an increased collection of 3D-FSE images. This will primarily focus on improving both size and quality of the data, thereby enriching the resources available for this study. Another limitation of the concept of adding 3D-FSE to the segmentation model training process is the lack of access to 3D-FSE in all centers. However, as the utilization of this pulse sequence continues to expand, we anticipate including more 3D-FSE CET1 image sets in publicly available data sets. This would enable future studies to incorporate a larger and more diverse range of data, fostering improved understanding and performance of models in the field of medical imaging and analysis. An additional constraint of our study pertains to the generalizability of our 3D FSE model, which primarily applies to the specific acquisition protocols used in the Mayo Clinic.

## Conclusion

The study findings indicate that models trained on 3D-FSE images exhibit enhanced performance in segmenting CEL across both 3D-FSE and IR-GRE image sets. The optimization and refinement of GBM segmentation models can be achieved incorporating 3D-FSE image sets through a fine-tuning step, leading to improved performance. In future endeavors, we aim to expand our data set with additional 3D-FSE images, focusing on enhancing the size of data available for this research.

## Potential Competing Interests

Given his role as Editorial Board Member, Dr Bradley Erickson had no involvement in the peer-review of this article and had no access to information regarding its peer-review.
